# Current Evidence regarding Prophylactic Antibiotics in Head and Neck and Maxillofacial Surgery

**DOI:** 10.1155/2014/879437

**Published:** 2014-07-08

**Authors:** Kilian Kreutzer, Katharina Storck, Jochen Weitz

**Affiliations:** ^1^Department of Oral and Maxillofacial Surgery, Technische Universität München, Klinikum rechts der Isar, Ismaninger Straße 22, 81675 Munich, Germany; ^2^Department of Otorhinolaryngology Head and Neck Surgery, Technische Universität München, Klinikum rechts der Isar, Ismaninger Straße 22, 81675 Munich, Germany

## Abstract

Antibiotic prophylaxis is commonly used to decrease the rate of infections in head and neck surgery. The aim of this paper is to present the available evidence regarding the application of antibiotic prophylaxis in surgical procedures of the head and neck region in healthy patients. A systemic literature review based on Medline and Embase databases was performed. All reviews and meta-analyses based on RCTs in English from 2000 to 2013 were included. Eight out of 532 studies fulfilled all requirements. Within those, only seven different operative procedures were analyzed. Evidence exists for the beneficial use of prophylactic antibiotics for tympanostomy, orthognathic surgery, and operative tooth extractions. Unfortunately, little high-level evidence exists regarding the use of prophylactic antibiotics in head and neck surgery. In numerous cases, no clear benefit of antibiotic prophylaxis has been shown, particularly considering their potential adverse side effects. Antibiotics are often given unnecessarily and are administered too late and for too long. Furthermore, little research has been performed on the large number of routine cases in the above-mentioned areas of specialization within the last few years, although questions arising with respect to the treatment of high-risk patients or of specific infections are discussed on a broad base.

## 1. Introduction

With a focus on perioperative antibiotic regimes, the head and neck region attracts attention because bacterial colonization is omnipresent and the number of severe postoperative infections is comparatively low in the healthy patient. The latter is not surprising considering the superior immunologic structures and blood supply of the tissues in this region. Whereas postoperative surgical site infection (SSI) is rare in patients undergoing clean head and neck operations [1], surgeons often aim to initiate proper wound healing by prophylactic perioperative antibiotics in “clean”-contaminated sites. In these cases, the aim is to minimize the perioperative bacterial load to a level that will not lead to clinical infection [2]. Hence, a close look on the current evidence regarding prophylactic perioperative antibiotic regimes in common surgery seems to be appropriate.

The regular questioning of the benefit of general prophylactic antibiosis is an important issue. Because of the anxiety with respect to postoperative complications, especially in elective surgery, many surgeons tend to prescribe antibiotics thereby neglecting the adverse side effects of this medication. Furthermore, such treatment might support the development of antibiotic-resistant colonies and might involve unnecessary expenditure for the individual or for the health care system. In return, a higher level of safety for the well-being of the patient and the outcome of the operation is expected [3–5].

Thus, the general question needs to be raised as to whether antibiotic prophylaxis is necessary in surgery. Bowater et al. have compiled three conclusions in a meta-analysis of randomized controlled trails (RCTs) demonstrating the challenge of this topic. First, the use of an antibiotic prophylaxis cannot be substantiated because of the variety of surgical procedures. Second, the risk of SSI is reduced by the administration of antibiotics, even if this has not been established by rigorous study. Third, the use of antibiotics should only be omitted if they are demonstrated to lack any value [6].

This paper gives a brief overview of the current evidence based recommendations regarding antibiotic prophylaxis for elective and emergency procedures in the noninfected operating field in the healthy patient. In addition, we point out those interventions in which antibiotic prophylaxis can be omitted with a clear conscience.

## 2. Materials and Methods

### 2.1. Study Design

To highlight the current evidence for prophylactic antibiotics in head and neck and maxillofacial surgery, a comprehensive review of the literature was undertaken.

### 2.2. Article Selection

For the study, an EMBASE (Elsevier Life Science Solutions) and MEDLINE (Pubmed) search was performed. The “Medical Subject Headings” terms were “prophylactic antibiotics” and “head and neck surgery” or “maxillofacial surgery.” We included all relevant studies in English from 2000 to 2013 (see Table 1).

**Figure 1:**
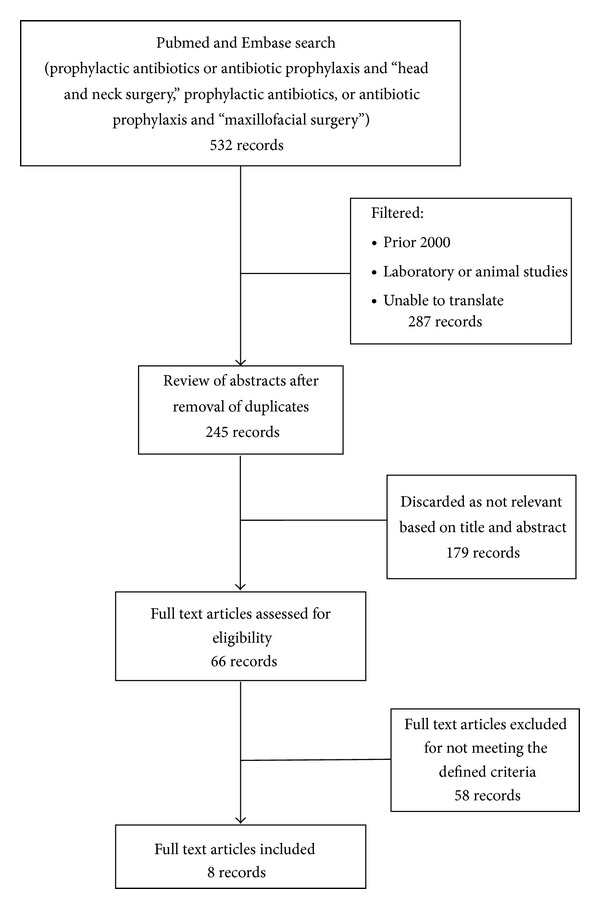


We excluded case reports, technical notes, animal or laboratory studies, expert opinions, tutorials, nonsystematic reviews, and neurosurgical case load. Furthermore, studies involving antibiotic prophylaxis in patients who underwent chemotherapy (CTx) or radiotherapy (RTx) or who had a clinical infection or in an immunocompromised (ICP) status were not taken into account (see Figure 1).

## 3. Results

See Table 2.

### 3.1. Tooth Extraction and Osteotomies

In 2012, a Cochrane review was published concerning third molar extractions. In 18 double-blind placebo controlled trails, a placebo was compared with perioperative antibiotics in third molar removal in 2456 cases. Even though benefits could be identified with regard to the reduction of infections (NNT = 12) and alveolitis sicca (NNT = 38), the authors mentioned that, because of adverse effects in 1 of 21 cases, antibiotic prophylaxis was not indicated in a healthy patient. No influence of whether the antibiotics that were given pre-, peri-, or postoperative were seen in the rate of SSIs [7].

### 3.2. Insertion of Dental Implants

Bacterial contamination at the time of implant insertion is considered to be the origin of postoperative infection or even long-term implant loss. In a systematic review Esposito et al. demonstrated that the preoperative administration of 2 g amoxicillin prevented the failure of dental implants within the first three months in 1 of 33 cases. No statistically significant differences in postoperative infections and adverse events were observed. Four RCTs with a total of 1007 implant insertions in healthy patients were included in the review. No evidence was found for the use of postoperative antibiotics [8]. If intraoral bone grafting procedures are required prior to dental implantation, an antibiotic perioperative prophylaxis is recommended for all patients [9–11]. No significant influence was observed with regard to the type, dosage, or period of antibiotic; surprisingly, the duration of operation could not be identified as a significant factor for infection [11].

### 3.3. Orthognathic Surgery

Up to the 1970s, some authors considered that antibiotics should not be used routinely in sagittal osteotomy/orthognathic surgery [12]. Danda evaluated 8 studies comparing short versus extended postoperative antibiotics in a meta-analysis. Patients who received antibiotic prophylaxis with short-term postoperative antibiotics had a significant higher risk of wound infection compared with patients receiving extended-term postoperative antibiotics (frequency: short-term 11.2% versus extended-term 3.8%; *x* ^2^ = 9.453; *P* < 0.01). On average, 13.5 patients had to be treated with antibiotics to prevent one case of wound infection (NNT = 13.5) [13]. Tan et al. compared intravenous versus oral application of postoperative antibiotics in a randomized clinical trial with 42 patients and found no statistically significant difference in the infection rate [14].

### 3.4. Maxillofacial Trauma

For the most common fracture in the maxillofacial area, namely, mandible fractures, many RCTs and retrospective case series have been published over the last few years. In a systematic review in 2011, Kyzas [15] analyzed 31 studies with 5437 patients regarding the benefit of antibiotic prophylaxis for avoiding SSIs. However, important information is lacking concerning time between injury and treatment, the type of applied antibiotic agent, the route of administration, and the duration and dosage of the antibiotics used in the majority of studies. The evidence to support the use of antibiotic prophylaxis is limited and of low quality. Nevertheless, a few pointers suggest that prophylactic antibiosis might be better than nothing in the prevention of SSIs. Those authors who recommend antibiotic prophylaxis for open reduction and internal fixation of compound mandibular fractures point out that the duration of antibiotics should not be more than 24 hours [9, 16, 17].

Andreasen et al. have reviewed four randomized studies concerning the potential benefit of antibiotic prophylaxis in maxillofacial fracture treatment [18] including zygoma, maxilla, condyle, and mandible.

He concludes that the administration of antibiotics results in a significant reduction in postinjury infections by threefold. Interestingly, no infection has been found in the zygoma, maxilla, or condylar region, irrespective of antibiotic prophylaxis.

### 3.5. Tonsillectomy

Only one review matching the inclusion criteria was identified. Performing a Cochrane review based on 10 randomized controlled trails concerning the effect of prophylactic antibiotics for posttonsillectomy morbidity, Dhiwakar et al. described three primary outcomes, namely, pain, consumption of analgesia, and secondary hemorrhage, plus three secondary outcomes, namely, fever, time taken to resume normal diet and activities, and adverse events [19]. The results were evaluated on the basis of fever being a clinical sign of SSI. A meta-analysis of two studies revealed that antibiotics reduced the number of patients manifesting fever (RR with antibiotics 0.63, 95% CI 0.46 to 0.85, *P* = 0.002). Most RCTs acknowledge a positive and statistically significant effect attributable to the administration of prophylactic antibiotics on the appearance of postoperative fever. The authors concluded that antibiotics might reduce fever [19].

### 3.6. Endoscopic Sinus Surgery

Saleh et al. evaluated the use of antibiotic prophylaxis in endoscopic sinus surgery. In a systematic literature search, 4 RCTs could be included, and a meta-analysis of three RCTs was conducted. No evidence for a statistically significant reduction in the incidence of infections after endoscopic sinus surgery was found (RR 0.76, 95% CI 0.64 to 0.09). The authors concluded that the current evidence did not support the routine use of prophylactic postoperative antibiotics in endoscopic sinus surgery [20].

### 3.7. Tympanostomy

In a meta-analysis by Hochman et al., the effect of topical antibiotics after tympanostomy was assessed. Nine studies with a total of 716 ears and 1344 patients were taken into account. The authors concluded that topical antibiotics were able to reduce the incidence of posttympanostomy otorhea as a sign of SSI by about 48% (OR 0.518; 95% CI 0.39–0.69; *P* value 0.000). In this meta-analysis, a statistically significant benefit was seen in the collective result [21].

## 4. Discussion

In addition to the achievement of the expected operative result, the prevention of complications is the most important cofactor for surgical success. SSI is considered to be one of the most severe complications during surgical follow-up care. Our aim has been to find the best evidence for or against prophylactic antibiotics in common procedures in head and neck and maxillofacial surgery. However, only a few reviews or meta-analyses with a high level of evidence regarding the topics above have been identified in our systematic review.

The oral cavity is defined as a clean-contaminated site. Whereas many authors support antibiotic prophylaxis even for otherwise healthy patients, many other reviewers perceive no evidence for its use, for example, in teeth extractions, even though these are one of the most frequently performed operations in the head and neck region [7, 22, 23]. Recommendations concerning antibiotic prophylaxis for standard tooth extractions should be regarded highly critically, as most studies observe wound infection only after the removal of third molars, which are often impacted and seldom infected or highly carious. Hence, clear evidence is lacking for antibiotic prophylaxis in tooth removal attributable to caries or periodontal impairment.

As implants have become increasingly important for replacing missing teeth, several studies deal with the effect of antibiotics on wound infection and early implant failure.

Even though prophylactic antibiotics given orally 1 h preoperatively significantly reduce early dental implant failure, no statistically significant differences in postoperative infections and adverse events have been observed [8]. As no major adverse events have been reported, the routine use of a single dose of amoxicillin (2 g) can be considered just before implant placement. If intraoral bone grafting is required prior to implantation, an antibiotic perioperative prophylaxis is recommended for all patients [9–11], even though no studies with a high level of evidence exist for this procedure.

For maxillofacial trauma, especially mandible fractures, many randomized and retrospective studies have been performed to evaluate the effect of antibiotic prophylaxis to minimize SSI. Unfortunately, important information is lacking concerning the type, duration, dosage, and route of administration of the applied antibiotic agent and the time between injury and definite treatment. For example, two clinical randomized studies have compared different antibiotic regimes with no control groups [24, 25]. Not a single study presents NNT, and many RCTs do not ensure allocation concealment and inadequately describe the mode of randomization [15]. This makes it difficult to give clear recommendations for antibiotic use. Nonetheless, a few pointers suggest that antibiotic prophylaxis might be better than nothing in preventing SSIs [15, 18]. Practically no data exists regarding the effect of antibiotics on other maxillofacial fractures such as zygoma, condyle, or maxilla, in particular because of the smaller number of cases and the rareness of SSIs in these procedures.

However, the wide spread use of antibiotics in the treatment of closed fractures of the central midface and the ascending ramus of the mandible is declining; in contrast to the recommendations given during previous decades, the tendency nowadays is to support the use of antibiotic prophylaxis in orthognathic surgery [12, 16–18]. In particular, the extended duration of antibiotic prophylaxis seems to reduce the risk of SSIs significantly, whereas its dosage form (oral versus intravenous) is negligible [13, 14].

Because of the demand for a nearly perfect aesthetic outcome in the young cleft patient with little tolerance concerning infections that increase the risk of wound breakdown, palatal fistulas, poor speech or growth, and aesthetic results [26], a large number of patients might be receiving antibiotics when no clear indication exists for such prophylaxis [27]. Increasing evidence suggests that the application of nonindicated antibiotics exposes the young patients to the unnecessary risk of adverse effects and antibiotic-associated complications such as Clostridium difficile infection [28, 29].

Unfortunately the interesting systematic literature review by Russell and Goldberg [30], which includes five randomized controlled trails in order to establish guidelines for the use of prophylactic antibiosis in clean-contaminated oncologic head and neck surgery, lacks a control group and has had to be excluded from this review. In clean head and neck tumor surgery, antibiotic prophylaxis should be considered [9] but is not indicated [2]. Whereas the isolated neck dissection is considered to be clean surgery, the often unavoidable expansion of the operation site to the clean-contaminated intraoral and pharyngeal region is characteristic for tumor surgery of the head and neck region. In these cases, antibiotic prophylaxis is effective and is administered prior to the start of surgery [30]. No evidence has been found to support the use of antibiotic prophylaxis beyond 24 h postoperatively [2]. On comparing 1-day versus 5-day prolonged prophylaxis, no advantage has been seen, even when regional flap or free-flap reconstruction is performed [31]. The evidence for antibiotic prophylaxis is most critical in clean and benign head and neck surgery. SSIs occur only in <1% of patients undergoing this kind of treatment, and hence, in these cases, antibiotic prophylaxis is not thought to be beneficial [1, 32]. On the other hand, the recommendation for antibiotic prophylaxis of clean-contaminated head and neck surgery is based on strong evidence [9, 33]. This includes ablative tumor surgery, which usually involves tunneling for placing flaps into the oral cavity or the pharynx for reconstruction.

Even though tonsillectomy is one of the most routine procedures in head and neck surgery, we have found only one meta-analysis dealing with the effect of prophylactic antibiotics [19]. Not explicitly stating SSI as an outcome, the secondary outcome fever was taken in account. The reduction of postoperative fever was significant in some studies, whereas no evidence for the use of antibiotics could be found in others. The effect can be explained on the basis of the containment of bacteraemia during and immediately after tonsillectomy. However, the authors see the main limitation of their review in the weak methodology of the included trails. In conclusion, they decline the routine administration of antibiotics to all patients undergoing tonsillectomy.

With regard to endoscopic sinus surgery, Saleh et al. [20] have been unable to demonstrate a statistically significant reduction in infection by the use of antibiotic prophylaxis. Based on studies with a high level of evidence, the authors consider the main limitation to be the small number of patients and studies. The antibiotics used were cefuroxime and amoxicillin/clavulanate. In one study, the comparison group received the same antibiotic as the single preoperative dose. Nevertheless, this meta-analysis comes out strongly against the use of prophylactic antibiotics in endoscopic sinus surgery.

The most significant advocate for the use of antibiotic prophylaxis in the field of head and neck surgery was found in the avoidance of posttympanostomy tube otorrhea. The meta-analysis by Hochman et al. [21] demonstrates that incidence can be reduced by about 50%. However, the studies involved can be split up into two subgroups. Three studies examined the cases “by ear,” while six studies involved data “by patient.” When considered independently in the “by ear” studies, no statistical significance could be found. The authors acknowledge that this conflicting information might depend more on the inclusion criteria, rather than the data. The conclusion that they derive from their study is that a practitioner should offer antibiotic drops to all patients receiving tympanostomy.

Unfortunately, the current evidence extracted by this systematic review does not allow broad conclusions on the use of prophylactic antibiotics in clean or clean-contaminated head and neck surgery. Moreover, the topic of the prophylactic antibiotic regime in benign and malign tumor surgery in this region of specialization has not been challenged satisfactory. Furthermore, little research with strong evidence has been performed on the large number of routine cases in the above-mentioned regions of specialization within the last few years, whereas questions arising from the treatment of high-risk patients or of specific infections are merely discussed on a broad base. We should also emphasize that not all of the discussed recommendations are appropriate for patients at risk of developing severe infections because of immunodeficiency, radiation therapy, or chemotherapy or who are in need of endocarditis prophylaxis.

## 5. Conclusion

The fear of SSIs is the motivation for the use of antibiotics in noninfected sites in clean or clean-contaminated surroundings. In this systematic literature review, only seven procedures for head and neck surgery or maxillofacial surgery could be identified that had been reviewed on an adequate level of evidence. Evidence exists for the beneficial use of antibiotics in tympanostomy, orthognathic surgery, and operative tooth extractions. However, because of their adverse side effects, no recommendations are made for the use of antibiotics in the last-mentioned procedure. In conclusion, we have found a lack of RCT based reviews and meta-analyses dealing with the question of prophylactic antibiotics in head and neck and maxillofacial surgery.

## Figures and Tables

**Table 1:** 

Inclusion criteria	Exclusion criteria
Pre-/peri-/postoperative administration of prophylactic antibiotics Systematic reviews and meta-analyses Publication date 2000–2013 Language: English Human studies Outcome: SSI	Case reports, technical notes, expert opinions, tutorials, nonsystematic reviews, and RCTs Animal or laboratory studies Neurosurgical case load High-risk patients (RTx, CTx, and ICP) Therapeutic administration of antibiotics

**Table 2:** 

Author	Year	Study design	Procedure	Sample size	SSI	*P* value	ARR	NNT
Andreasen et al. [18]	2006	Systematic review	Maxillofacial fractures	4 Studies				

Lawler et al. [34]	2005	Review	Dentoalveolar surgery	4 studies				

Lodi et al. [7]	2012	Interventional review	Third molars	18 studies with 2456 participants	Compared with placebo, antibiotics probably reduce the risk of infection in patients undergoing third molar extraction(s) by approximately 70%.	SSI: *P* < 0.0001 Dry socket: *P* = 0.03	SSI: 0.29 (95% CI 0.16 to 0.50) Dry socket: 0.62 (95% CI 0.41 to 0.95)	SSI: 12 Dry socket 38

Abubaker and Rollert [25]	2001	RCT	Mandible fractures	30 patients (Group 1: 14, Group 2: 16). All patients received penicillin 2 M i.v. every 4 hours through the pre- and intraoperative period and for 12 hours postoperatively Group 1 then received 500 mg penicillin VK every 6 hours for 5 days, group 2 placebo for same duration and under the same schedule	Group 1: 2/14 (14.3%) Group 2: 2/16 (12.5%)	*P* = 0.01 No significant difference		

Esposito et al. [8]	2010	Intervention review Cochrane	Dental implantation	6 RCTs, *n* = 1162	The meta-analyses of the six trials showed a statistically significant higher number of participants experiencing implant failures in the group not receiving antibiotics	*P* = 0.002	0.33	25

Hochman et al. [21]	2006	Meta-analysis	Tympanostomy	9 RCTs, *n* = 2060	Topical prophylactic antibiotic drops for at least 48 h postoperatively	*P* = 0.000		2

Dhiwakar et al. [19]	2012	Cochrane review	Tonsillectomy	10 RCT, antibiotics to reduce posttonsillectomy morbidity	Fever as secondary outcome	*P* = 0.002	0.63, (95% CI 0.46 to 0.85)	

Saleh et al. [20]	2012	Systematic review and meta-analysis	Endoscopic sinus surgery	Total of 4 RCT and meta-analyses with 3 RCT; *n* = 492			0.76, 95% CI 0.64 to 0.09	

## References

[1] Johnson JT, Wagner RL (1987). Infection following uncontaminated head and neck surgery. *Archives of Otolaryngology—Head and Neck Surgery*.

[2] Koshkareva YA, Johnson JT (2014). What is the perioperative antibiotic prophylaxis in adult oncologic head and neck surgery?. *The Laryngoscope*.

[3] Liu S, Chiu Y, Lin W, Chen S (2008). Using information technology to reduce the inappropriate use of surgical prophylactic antibiotic. *European Archives of Oto-Rhino-Laryngology*.

[4] Goldstein EJC, Citron DM, Merriam CV, Abramson MA (2009). Infection after elective colorectal surgery: bacteriological analysis of failures in a randomized trial of cefotetan vs. ertapenem prophylaxis. *Surgical Infections*.

[5] Schwartz B, Bell DM, Hughes JM (1997). Preventing the emergence of antimicrobial resistance: a call for action by clinicians, public health officials, and patients. *Journal of the American Medical Association*.

[6] Bowater RJ, Stirling SA, Lilford RJ (2009). Is antibiotic prophylaxis in surgery a generally effective intervention?: testing a generic hypothesis over a set of meta-analyses. *Annals of Surgery*.

[7] Lodi G, Figini L, Sardella A, Carrassi A, Del Fabbro M, Furness S (2012). Antibiotics to prevent complications following tooth extractions. *The Cochrane Database of Systematic Reviews*.

[8] Esposito M, Worthington HV, Loli V, Coulthard P, Grusovin MG (2010). Interventions for replacing missing teeth: antibiotics at dental implant placement to prevent complications.. *Cochrane Database of Systematic Reviews*.

[9] Brown K, de Beaux A, Qureshi S, Twaddle S (2008). Antibiotic prophylaxis in surgery. *Scottish Intercollegiate Guidelines Network*.

[10] Lindeboom JA, van den Akker HP (2003). A prospective placebo-controlled double-blind trial of antibiotic prophylaxis in intraoral bone grafting procedures: a pilot study. *Oral Surgery, Oral Medicine, Oral Pathology, Oral Radiology and Endodontics*.

[11] Lindeboom JA, Frenken JW, Tuk JG, Kroon FH (2006). A randomized prospective controlled trial of antibiotic prophylaxis in intraoral bone-grafting procedures: preoperative single-dose penicillin versus preoperative single-dose clindamycin. *International Journal of Oral and Maxillofacial Surgery*.

[12] Peterson LJ, Booth DF (1976). Efficacy of antibiotic prophylaxis in intraoral orthognathic surgery. *Journal of Oral Surgery*.

[13] Danda AK, Ravi P (2011). Effectiveness of postoperative antibiotics in orthognathic surgery: a meta-analysis. *Journal of Oral and Maxillofacial Surgery*.

[14] Tan SK, Lo J, Zwahlen RA (2011). Are postoperative intravenous antibiotics necessary after bimaxillary orthognathic surgery? A prospective, randomized, double-blind, placebo-controlled clinical trial. *International Journal of Oral and Maxillofacial Surgery*.

[15] Kyzas PA (2011). Use of antibiotics in the treatment of mandible fractures: a systematic review. *Journal of Oral and Maxillofacial Surgery*.

[16] Zix J, Schaller B, Iizuka T, Lieger O (2013). The role of postoperative prophylactic antibiotics in the treatment of facial fractures: a randomised, double-blind, placebo-controlled pilot clinical study. Part 1: Orbital fractures in 62 patients. *The British Journal of Oral and Maxillofacial Surgery*.

[17] Schaller B, Soong PL, Zix J, Iizuka T, Lieger O (2013). The role of postoperative prophylactic antibiotics in the treatment of facial fractures: a randomized, double-blind, placebo-controlled pilot clinical study—part 2: mandibular fractures in 59 patients. *British Journal of Oral and Maxillofacial Surgery*.

[18] Andreasen JO, Jensen SS, Schwartz O, Hillerup Y (2006). A systematic review of prophylactic antibiotics in the surgical treatment of maxillofacial fractures. *Journal of Oral and Maxillofacial Surgery*.

[19] Dhiwakar M, Clement WA, Supriya M, McKerrow W (2012). Antibiotics to reduce post-tonsillectomy morbidity. *Cochrane Database of Systematic Reviews*.

[20] Saleh AM, Torres KM, Murad MH, Erwin PJ, Driscoll CLW (2012). Prophylactic perioperative antibiotic use in endoscopic sinus surgery: a systematic review and meta-analysis. *Otolaryngology—Head and Neck Surgery*.

[21] Hochman J, Blakley B, Abdoh A, Aleid H (2006). Post-tympanostomy tube otorrhea: a meta-analysis. *Otolaryngology: Head and Neck Surgery*.

[22] Susarla SM, Sharaf B, Dodson TB (2011). Do antibiotics reduce the frequency of surgical site infections after impacted mandibular third molar surgery?. *Oral and Maxillofacial Surgery Clinics of North America*.

[23] Halpern LR, Dodson TB (2007). Does prophylactic administration of systemic antibiotics prevent postoperative inflammatory complications after third molar surgery?. *Journal of Oral and Maxillofacial Surgery*.

[24] Heit JM, Stevens MR, Jeffords K (1997). Comparison of ceftriaxone with penicillin for antibiotic prophylaxis for compound mandible fractures. *Oral Surgery, Oral Medicine, Oral Pathology, Oral Radiology, and Endodontics*.

[25] Abubaker AO, Rollert MK (2001). Postoperative antibiotic prophylaxis in mandibular fractures: a preliminary randomized, double-blind, and placebo-controlled clinical study. *Journal of Oral and Maxillofacial Surgery*.

[26] Chuo CB, Timmons MJ (2005). The bacteriology of children before primary cleft lip and palate surgery. *Cleft Palate-Craniofacial Journal*.

[27] Rangel SJ, Fung M, Graham DA, Ma L, Nelson CP, Sandora TJ (2011). Recent trends in the use of antibiotic prophylaxis in pediatric surgery. *Journal of Pediatric Surgery*.

[28] Donskey CJ, Chowdhry TK, Hecker MT (2000). Effect of antibiotic therapy on the density of vancomycin-resistant enterococci in the stool of colonized patients. *The New England Journal of Medicine*.

[29] Samonis G, Gikas A, Anaissie EJ (1993). Prospective evaluation of effects of broad-spectrum antibiotics on gastrointestinal yeast colonization of humans. *Antimicrobial Agents and Chemotherapy*.

[30] Russell MD, Goldberg AN (2012). What is the evidence for use of antibiotic prophylaxis in clean-contaminated head and neck surgery?. *The Laryngoscope*.

[31] Reiffel AJ, Kamdar MR, Kadouch DJM, Rohde CH, Spector JA (2010). Perioperative antibiotics in the setting of microvascular free tissue transfer: Current practices. *Journal of Reconstructive Microsurgery*.

[32] Simo R, French G (2006). The use of prophylactic antibiotics in head and neck oncological surgery. *Current Opinion in Otolaryngology & Head and Neck Surgery*.

[33] Velanovich V (1991). A meta-analysis of prophylactic antibiotics in head and neck surgery. *Plastic and Reconstructive Surgery*.

[34] Lawler B, Sambrook PJ, Goss AN (2005). Antibiotic prophylaxis for dentoalveolar surgery: is it indicated?. *Australian Dental Journal*.

